# The prognostic value of a seven-lncRNA signature in patients with esophageal squamous cell carcinoma: a lncRNA expression analysis

**DOI:** 10.1186/s12967-020-02224-z

**Published:** 2020-01-31

**Authors:** Nuo-Qing Weng, Jun Chi, Jing Wen, Shi-Juan Mai, Mei-Yin Zhang, Long Huang, Ji Liu, Xian-Zi Yang, Guo-Liang Xu, Jian-Hua Fu, Hui-Yun Wang

**Affiliations:** 1 State Key Laboratory of Oncology in South China, Collaborative Innovation Center for Cancer Medicine, Sun Yat-Sen University Cancer Center, 651 Dongfeng East Road, Building 2, Room 704, Guzngzhou, 510060 China; 2 Guangdong Esophageal Cancer Institute, Guangzhou, 510060 China; 3 Department of Endoscopy and Laser, Sun Yat-Sen University Cancer Center, Guangzhou, 510060 China; 4 Department of Thoracic Oncology, Sun Yat-Sen University Cancer Center, Guangzhou, 510060 China; 5 Department of Oncology, The Second Affiliated Hospital of Nanchang University, Nanchang, China

**Keywords:** Esophageal cancer, lncRNA, Expression profile, Survival, TNM stage

## Abstract

**Background:**

Long non-coding RNAs (lncRNAs) have been reported to be prognostic biomarkers in many types of cancer. We aimed to identify a lncRNA signature that can predict the prognosis in patients with esophageal squamous cell carcinoma (ESCC).

**Methods:**

Using a custom microarray, we retrospectively analyzed lncRNA expression profiles in 141 samples of ESCC and 81 paired non-cancer specimens from Sun Yat-Sen University Cancer Center (Guangzhou, China), which were used as a training cohort to identify a signature associated with clinical outcomes. Then we conducted quantitative RT-PCR in another 103 samples of ESCC from the same cancer center as an independent cohort to verify the signature.

**Results:**

Microarray analysis showed that there were 338 lncRNAs significantly differentially expressed between ESCC and non-cancer esophagus tissues in the training cohort. From these differentially expressed lncRNAs, we found 16 lncRNAs associated with overall survival (OS) of ESCC patients using Cox regression analysis. Then a 7-lncRNA signature for predicting survival was identified from the 16 lncRNAs, which classified ESCC patients into high-risk and low-risk groups. Patients with high-risk have shorter OS (HR: 3.555, 95% CI 2.195–5.757, p < 0.001) and disease-free survival (DFS) (HR: 2.537, 95% CI 1.646–3.909, p < 0.001) when compared with patients with low-risk in the training cohort. In the independent cohort, the 7 lncRNAs were detected by qRT-PCR and used to compute risk score for the patients. The result indicates that patients with high risk also have significantly worse OS (HR = 2.662, 95% CI 1.588–4.464, p < 0.001) and DFS (HR 2.389, 95% CI 1.447–3.946, p < 0.001). The univariate and multivariate Cox regression analyses indicate that the signature is an independent factor for predicting survival of patients with ESCC. Combination of the signature and TNM staging was more powerful in predicting OS than TNM staging alone in both the training (AUC: 0.772 vs 0.681, p = 0.002) and independent cohorts (AUC: 0.772 vs 0.660, p = 0.003).

**Conclusions:**

The 7-lncRNA signature is a potential prognostic biomarker in patients with ESCC and may help in treatment decision when combined with the TNM staging system.

## Background

Esophageal cancer (EC) is the eighth most common cancer worldwide, with poor overall 5-year survival rate ranging from 15 to 25% [1]. EC has two main subtypes: esophageal squamous cell carcinoma (ESCC) predominantly found in Asia, Africa, and South America, and esophageal adenocarcinoma (EAC) predominant in North America and Europe [2]. ESCC accounts approximately 80% of the EC worldwide, and is a highly aggressive squamous cell carcinoma [3]. Regarding the etiology, the incidence of ESCC is closely related with alcohol and tobacco [4]. The primary method for the diagnosis of EC patients is endoscopy with biopsy [5]. Currently, prognosis and treatment of EC are mainly based on the TNM staging system. However, ESCC patients with same M0 stage according to the 7th/8th edition AJCC staging system have distinct prognoses [6]. Therefore, clinical TNM staging system needs further improvement [7]. Molecular biomarkers have proven to be of great prognostic value for cancer patients [8, 9]. However, no practical molecular biomarker has been used in the prognostication of EC patients yet, although various biomarkers have been reported in ESCC patients with the rapid development in the molecular biology [10–13]. Clinically, the main treatment options for EC patients include surgery with or without neoadjuvant therapy, and chemoradiotherapy with or without salvage surgery [14]. At present, there is no targeted therapy for EC patients, which has already been applied in patients with other types of cancer [15, 16]. Although many advances in treatment have been made, the survival of ESCC patients is still disappointedly low. Therefore, there is an urgent need to better understand the biology of ESCC and to identify other more potential molecular biomarkers or targets for prognosis and targeted therapy in the patients.

In recent decades, there have been numerous reports on coding-protein genes as biomarker for prediction of prognosis of ES patients [17–19], but none of them have been used in clinical practice. In the past few years, long non-coding RNAs (lncRNAs), defined as transcripts longer than 200 nt lacking protein-coding capacity, have been reported to have complex biological functions [20–22] and involved in tumorigenesis and metastasis [23]. Some research groups have reported that many single lncRNAs can be used as biomarkers for prediction of survival in ESCC patients [11], but there is only one study on lncRNA expression profile with microarray to date, which identified a prognostic signature (3-lncRNA) in ESCC patients [24]. Therefore, more high-throughput studies are needed to fully discover potential prognostic lncRNA signatures for ESCC patients. In this study, we aimed to explore the lncRNA expression profile in ESCC patients with a custom microarray and identify a lncRNA signature to predict the prognosis of ESCC patients, which may improve the TNM staging system.

## Methods

### Clinical specimens and study design

In this study, we first collected 141 esophageal squamous cell carcinoma (ESCC) specimens and 81 paired adjacent normal tissues (at least 2 cm away from the ESCC) from the tissue bank at Sun Yat-Sen University Cancer Center (Guangzhou, China). These samples were obtained from patients who underwent esophagectomy in the Cancer Center between Oct. 2007 and Sep. 2009, which were used as a training cohort. We then obtained another 103 ESCC samples from the patients who were received the esophagectomy between Mar. 2002 and Sep. 2009 at the Department of Thoracic Surgery in the same center, which were used as an independent cohort. The inclusion criteria for this study were as following: (i) pathologically confirmed ESCC; (ii) no pre-operative radiotherapy and/or chemotherapy; (iii) R0 resection; (iv) no patient died in 1 month after surgery; (v) available frozen tissue samples. The clinical characteristics of all patients in the two cohorts are listed in Table 1. The follow-up time ranged from 3 to 91 months with a median time of 37 months in the training cohort, and 1 month to 150 months with a median time of 43 months in the independent cohort. The overall survival (OS) was defined as the time from the date of surgery to the date of death or last follow-up, and the disease-free survival (DFS) was defined as the time from the date of surgery to the date of the recurrence in situ, distant metastasis, death or last follow-up. Tumor stage was determined according to the 7th edition of the TNM classification of the Union for International Cancer Control (UICC). This study was reviewed and approved by the Research Ethics Committee of Sun Yat-Sen University Cancer Center. Written informed consent forms were obtained from every patient prior to surgery.

**Table 1:** Clinical characteristics of ESCC patients in the training cohort and independent cohort

Characteristic	Training cohort	Independent cohort
Age		
≤ 59	66 (46.8%)	54 (52.4%)
> 59	75 (53.2%)	49 (47.6%)
Gender		
Male	112 (79.4%)	74 (71.8%)
Female	29 (20.6%)	29 (28.2%)
Tobacco use		
Yes	95 (67.4%)	66 (64.1%)
No	46 (32.6%)	37 (35.9%)
Alcohol use		
Yes	61 (43.3%)	40 (38.8%)
No	80 (56.7%)	63 (61.2%)
Tumor location		
Upper	13 (9.2%)	8 (7.8%)
Middle	95 (67.4%)	65 (63.1%)
Lower	33 (23.4%)	30 (29.1%)
Tumor size		
≤ 4	92 (67.3%)	63 (61.2%)
> 4	47 (33.3%)	40 (38.8%)
N stage		
N0	66 (46.8%)	64 (62.1%)
N1	45 (31.9%)	22 (21.4%)
N2	20 (14.2%)	13 (12.6%)
N3	10 (7.1%)	4 (3.9%)
TNM stage		
I	4 (2.8%)	10 (9.7%)
II	73 (51.8%)	57 (55.3%)
III	64 (45.4%)	36 (35.0%)

### RNA isolation, labeling and microarray hybridization

Total RNAs were isolated from fresh-frozen ESCC and noncancerous esophagus tissues using TRIzol reagent (Invitrogen) according to the manufacturer’s instructions, and the concentration and purity of total RNAs were estimated with a NanoDrop 2000 spectrophotometer (Thermo Scientific, Wilmington, DE, USA). If the ratio of A260/A280 was between 1.9 and 2.1, the total RNA was accepted for the subsequent experiments.

To fabricate the custom lncRNA microarray, we first selected 2412 lncRNAs from literatures and public databases for designing the probes. Then the lncRNA probes were synthesized by Invitrogen company (Shanghai, China). The probes were mixed well with printing buffer (1:1) and printed on the cleaned slides in SmartArray™ 136 printer (CapitalBio Inc., Beijing, China) as previously described [25, 26]. RNA labeling and microarray hybridization were conducted according to the published protocols [26, 27] with minor adjustments. Briefly, 2 μg of total RNAs from the training cohort were reverse transcribed using random primers and Cy5-dUTP or Cy3-dUTP with GoScript™ Reverse Transcription system (Promega) in a total reaction volume of 20 μl. The labeled cDNA products were purified and eluted in 100 μL, and thermally vacuum-evaporated to 25 μl. And then the labeled cDNAs were mixed with 25 μl hybridization solution containing 5× Denhart’s, 0.5% SDS and 3× SSC. After hybridization solution was added onto the microarray, hybridization was performed in a Hybridization Chamber (Corning Inc.) at a temperature of 45 °C for 16 to 18 h. After washing with consecutive solutions, the microarray was scanned by the LuxScan 10 K Microarray Scanner (CapitalBio, Beijing, China) at PMT 950. GenePix Pro 6.0 software (Axon Instruments, Foster City, CA, USA) was used to digitize the images.

Microarray raw data were subtracted by the background and normalized with quantile method [28], and then the normalized data were used for further analysis. The microarray data have been uploaded into the Gene Expression Omnibus Public Database at the National Center for Biotechnology Information, USA, and the accession number is GSE92986 (https://www.ncbi.nlm.nih.gov/geo/query/acc.cgi?acc=GSE92986).

### Real time quantitative reverse transcription-PCR

For the reverse transcription, total RNAs (2 μg) in a 20 μl volume were reversely transcribed with GoScript™ Reverse Transcription system (Promega). Then the quantitative PCR reaction was carried out with 1 μl of cDNA product, the specific primers and Platinum SYBR Green qPCR SuperMix-UDG reagents (Invitrogen, Carlsbad, CA, USA) using the following reaction cycle: 95 °C for 120 s, 45 cycles of 95 °C for 15 s, 60 °C for 60 s and a dissociation stage. The PCR primer sequences were listed in Additional file 1: Table S1, and the qPCR reaction for each sample was conducted in triplicate. The relative expression levels of lncRNAs were normalized by GAPDH (reference gene) expression and the median value of a given lncRNA’s expression in all samples, and presented as 2^−ΔΔCT^.

### Bioinformatic analysis

To investigate the biological functions of the 7 lncRNAs, we downloaded the 7 lncRNAs-related transcription factors (TF) from the published lncRNA-TF interactome database RegRNA2.0. Then, the target genes, which are transcribed by the TFs related to the seven lncRNAs, were downloaded from Harmonizome database. The Gene ontology (GO) and Kyoto Encyclopedia of Genes and Genomes (KEGG) pathway enrichment analysis were performed on these target genes to identify the biological processes associated with these seven lncRNAs. The clusterProfiler R package was used to perform the analysis and the P value less than 0.05 was defined as significant.

### Statistical analysis

Significance analysis of microarrays (SAM) program was conducted to identify differentially expressed lncRNAs between 141 samples of ESCC and 81 paired non-cancer specimens with a threshold of 1.25 fold change between the two groups with *P* values less than 0.1 in the training cohort. Univariate Cox regression analysis was used to identify the lncRNAs that were significantly associated with OS (*P* < 0.1) from the differentially expressed ones. Then we constructed a formula to calculate a risk score for each patients using all lncRNAs that were correlated with survival according to the method previously reported [29]: Risk score = $\sum\nolimits_{i = 1}^{n} {\left[ {{\text{Expression}}\left( {\text{n}} \right) \times {\text{Exp}}\left( {\text{Bn}} \right)} \right]}$. In this formula, the expression (n) is the expression value of every lncRNAs detected in each sample by microarray and the Exp(Bn) is the coefficient of each lncRNA obtained from Univariate Cox regression analysis. The risk score was calculated for each patients using the formula. The median value of the risk scores of all patients was employed to divide the patients into high-and low-risk groups in the training cohort. Kaplan–Meier method and log-rank test were employed to compare the survival of the two groups. Then we removed one lncRNA at a time from the survival lncRNAs (n) to compose n combinations (n-1 lncRNAs), and re-calculated the risk score for patients with all the combinations (risk score formulas), divided patients into high- and low-risk groups based on the risk score of each patient, and carried out the survival analysis again based on all the different lncRNA combinations. One lncRNA would be deleted if it is not a component of the best lncRNA combination (with the smallest p value in the survival analysis). Next, we composed n-1 combinations (n-2 lncRNAs) again and did the same analysis until 2-lncRNA combination. Finally, we compared all results and found the best combination (signature), which would be used for the further analysis.

χ2 test or Fisher’s exact test was applied to assess the relationship between lncRNA relative expression and clinical characteristics. The Kaplan–Meier method and the log-rank test were applied to assess the OS and DFS. The lncRNA signature, age, gender, pathologic grade, TNM stage, radiotherapy, chemotherapy, alcohol use, and tobacco use were analyzed as covariates with multivariate Cox regression by a forward LR approach in order to determine which feature(s) was an independent factor for OS and DFS. We then set up a new model that combined all the independent prognostic factors to predict the survival of each patient. Receiver operating characteristic (ROC) curves were employed to compare the sensitivity and specificity of TNM stage, lncRNA signature and the combination of both. Significance was defined as *p *< 0.05. The statistical analyses were done with SPSS version 23.0, GraphPad Prism 6.0c and MedCalc Version 11.4.2.

## Results

### LncRNA expression profiles of ESCC tissues

To profile lncRNA expression in ESCC tissues, we used the custom lncRNA microarray (Additional file 2: Fig. S1A) to detect 141 ESCCs and 81 paired esophagus tissues in the training cohort. After subtracting background and normalizing the hybridization intensity, we analyzed the lncRNA expression profile with SAM program. The result reveals that 338 lncRNAs are differentially expressed between ESCC and adjacent normal tissues (fold change > 1.25, p < 0.1). Of these 338 lncRNAs, 211 are upregulated and the other 127 are downregulated in ESCC tissues.

To validate lncRNA expression levels detected by the microarray analysis, we randomly selected two upregulated lncRNAs (ASLNC11164 and BQ376030) and two downregulated lncRNAs (XLOC_001061 and RP11-473M20.9) in ESCC tissues and detected them by quantitative RT-PCR in 40 pairs of ESCC and noncancerous esophagus tissues randomly chosen from the training cohort. As shown in Additional file 2: Fig. S1B, the expression levels of the 4 lncRNAs detected by qRT-PCR are consistent with those detected by microarray, suggesting that microarray data are repeatable and reliable and can be used for the further analysis.

### Identification of a 7-lncRNA signature for predicting the survival of ESCC patients in the training cohort

To explore the clinical role of lncRNA in ESCC patient prognosis, we first screened the 338 differentially expressed lncRNAs to find ones associated with survival using Univariate Cox regression analysis. With this analysis, we found 16 lncRNAs that were significantly associated with OS in the training cohort (Additional file 1: Table S2). Then we used the approach aforementioned in Method section to determine a lncRNA combination from the 16 lncRNAs, which can predict the survival of ESCC patients with highest efficiency. Finally, the best 7-lncRNA signature for predicting survival of patients was identified. The formula consists of a linear combination of the expression level of 7 lncRNAs weighted by Cox regression coefficient:

Risk score = (0.155 × expression value of BQ376030 + 0.112 × expression value of ASLNC11164 + 0.125 × expression value of BF894811 + 0.155 × expression value of RP11-473M20.9 − 0.182 × expression value of XLOC_007869 − 0.15 × XLOC_006476 − 0.172 × expression value of CK327190).

**Fig. 1:**
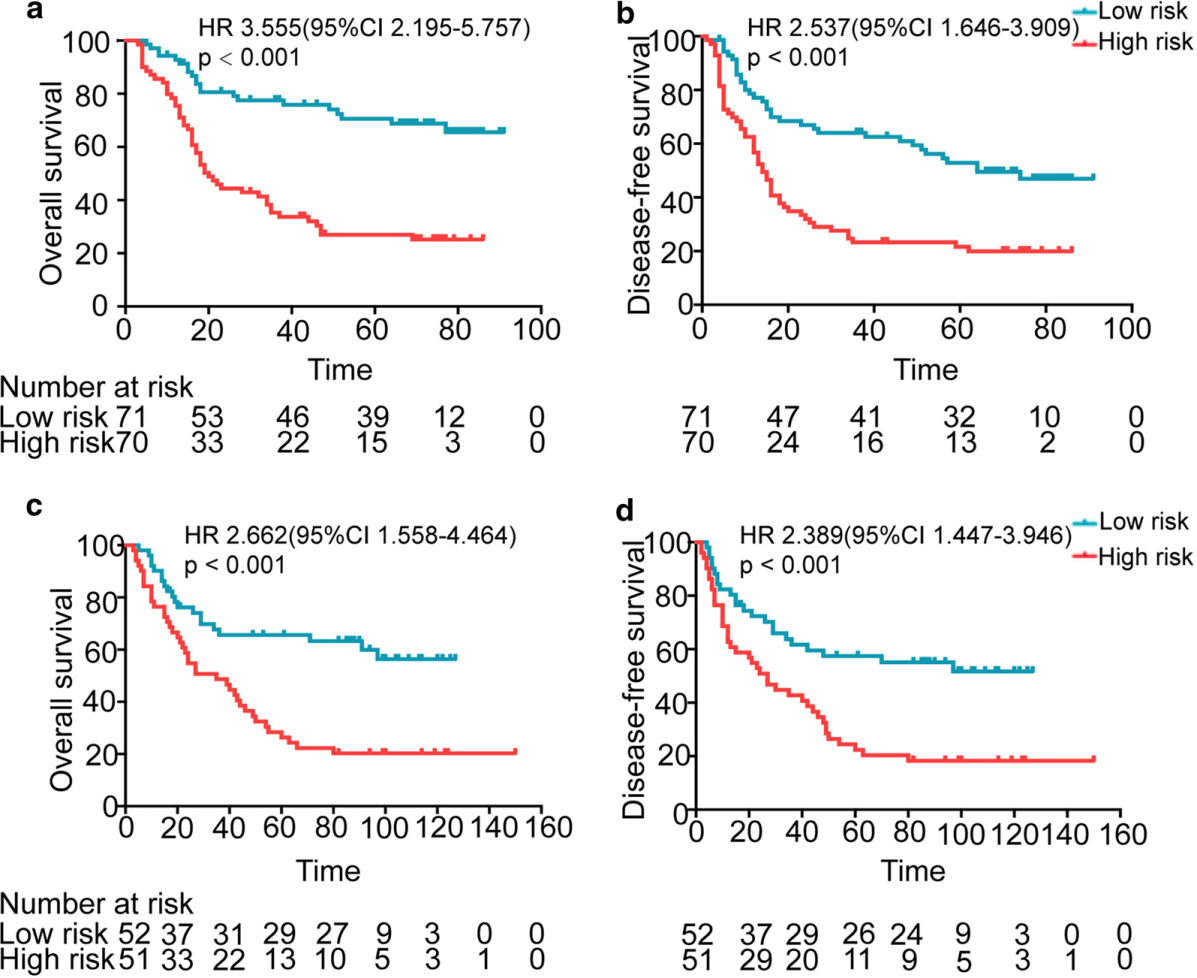
The 7-lncRNA signature is associated with survivals of ESCC patients in the training and independent cohorts. The risk score was calculated for each patient according to the 7-lncRNA signature and the patients were divided into a high- or low-risk group based on their risk score. Then Kaplan–Meier survival analysis was performed on the patients. **a** Overall survival (OS) curves of 141 patients with high-risk or low-risk in the training cohort. **b** Disease-free survival (DFS) curves of 141 patients in the training cohort. **c** OS curves of 103 patients with high-risk or low-risk in the independent cohort. **d** DFS curves of 103 patients with high-risk or low-risk in the independent cohort. Note: in the Kaplan–Meier survival curves, the survival time unit is months

Among these 7 lncRNAs, four are risk factors for survival and the other three are protective ones (Additional file 1: Table S2). The formula was employed to compute a risk score for each patient in the training cohort. With the risk score, patients were divided into high- and low-risk groups according to the median risk score in this cohort. Then we explored the relationship of the signature risk with clinical characteristics in ESCC patients, and found that patients with high risk had significantly higher lymph node metastasis rate and mortality rate (Table 2). Kaplan–Meier analysis revealed that patients in the high-risk group had a significantly shorter OS and DFS than those in the low-risk group (both *p* < 0.001, Fig. 1a, b).

**Table 2:** The relationships of 7-lncRNA signature and Clinical characteristics of ESCC patients in the training cohort and independent cohort

Characteristics	Training cohort (N = 141)		p value	Independent cohort (N = 103)		p value
	Low risk	High risk		Low risk	High risk	
	n (%)	n (%)		n (%)	n (%)	
Age						
≤ 59	37 (52.1%)	35 (50.0%)	0.802	29 (55.8%)	25 (49.0%)	0.493
> 59	34 (47.9%)	35 (50.0%)		23 (44.2%)	26 (51.0%)	
Gender						
Male	58 (81.7%)	54 (77.1%)	0.500	36 (69.2%)	38 (74.5%)	0.660
Female	13 (18.3%)	16 (22.9%)		16 (30.8%)	13 (25.5%)	
Tobacco use						
Yes	49 (69.0%)	46 (65.7%)	0.680	31 (59.6%)	35 (64.1%)	0.413
No	22 (31.0%)	24 (34.3%)		21 (40.4%)	16 (35.9%)	
Alcohol use						
Yes	34 (47.9%)	27 (38.6%)	0.260	22 (42.3%)	18 (35.3%)	0.546
No	37 (52.1%)	43 (61.4%)		30 (57.7%)	33 (64.7%)	
Tumor location						
Upper	6 (8.5%)	7 (10.0%)	0.510	2 (3.8%)	6 (11.8%)	0.210
Middle	51 (71.8%)	44 (62.9%)		32 (61.5%)	33 (64.7%)	
Lower	14 (19.7%)	19 (27.1%)		18 (34.6%)	12 (23.5%)	
Tumor size						
≤ 4	48 (67.6%)	46 (65.7%)	0.810	33 (63.5%)	30 (58.8%)	0.629
> 4	23 (32.4%)	24 (32.3%)		19 (36.5%)	21 (41.2%)	
N stage						
N0	40 (56.3%)	26 (37.1%)	0.050	35 (67.3%)	29 (56.9%)	0.240
N1	22 (31.0%)	23 (32.9%)		11 (21.2%)	11 (21.6%)	
N2	6 (8.5%)	14 (20.0%)		6 (11.5%)	7 (13.7%)	
N3	3 (4.2%)	7 (10.0%)		0 (0.0%)	4 (7.8%)	
TNM stage						
I	2 (2.81%)	2 (2.86%)	0.579	8 (15.4%)	2 (3.9%)	0.131
II	34 (47.9%)	39 (55.7%)		28 (53.8%)	29 (56.9%)	
III	35 (49.3%)	28 (40.0%)		16 (30.8%)	20 (39.2%)	
Survival status						
Survival cases	50 (70.4%)	20 (28.6%)	< 0.001	32 (61.5%)	11 (21.6%)	< 0.001
Death cases	21 (29.6%)	50 (71.4%)		20 (38.5%)	40 (78.4%)

### Confirmation of the 7-lncRNA prognostic signature in the independent cohort with qRT-PCR

In order to validate the reliability and repeatability of the 7-lncRNA prognostic signature in ESCC patients, we collected another 103 ESCC samples as a independent cohort in the same cancer center and extracted total RNA from these samples. The expression levels of the 7 lncRNAs were measured by qRT-PCR in these RNA samples. The qRT-PCR data were presented as 2^−ΔΔCT^ and analyzed with Cox regression. Then we constructed a new risk score formula with the same method used in the training cohort:

Risk score = (0.028 × expression value of BQ376030 + 0.053 × expression value of ASLNC11164 + 0.049 × expression value of BF894811 + 0.031 × expression value of RP11-473M20.9 − 0.058 × expression value of XLOC_007869 − 0.116 × XLOC_006476 − 0.061 × expression value of CK327190).

With this formula, we computed a risk score for every patient and divided the patients into low and high risk groups according to the median risk score in the independent cohort. Survival analysis revealed that high-risk patients showed notably poorer prognosis than low-risk ones in the independent cohort (both *P* < 0.001 in OS and DFS, Fig. 1c, d), suggesting that the 7-lncRNA signature is a reliable and repeatable survival predictor in patients with ESCC.

### The 7-lncRNA signature is an independent prognostic factor in patients with ESCC

**Fig. 2:**
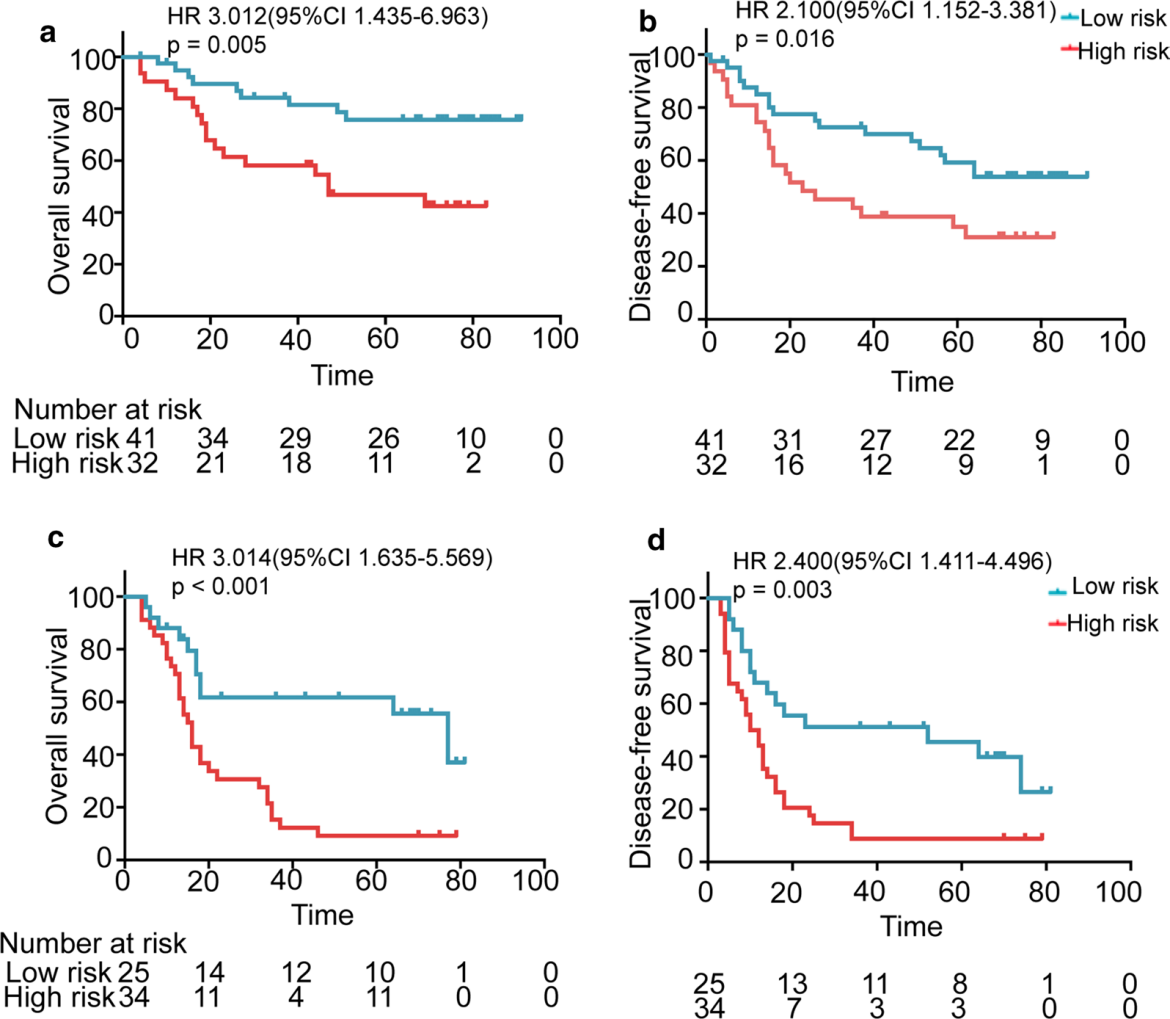
The 7-lncRNA signature can predict distinct survivals of ESCC patients with same TNM stage in the training cohort. The patients with same stage (II or III) were defined as high- or low-risk by the 7-lncRNA signature risk score and then analyzed with Kaplan–Meier survival curves. **a** Overall survival (OS) curves of 73 patients with high-risk or low-risk in the cases with TNM stage II in the training cohort. **b** Disease-free survival (DFS) curves in 73 patients with high-risk or low-risk in the cases with TNM stage II in the training cohort. **c** OS curves in 59 patients with high-risk or low-risk in the cases with TNM stage III in the training cohort. **d** DFS curves in 59 patients with high-risk or low-risk in the cases with TNM stage II in the training cohort. Note: in the Kaplan–Meier survival curves, the survival time unit is months

We then wanted know whether the 7-lncRNA signature was an independent predictor for patients with ESCC. To this end, we conducted univariate and multivariate Cox proportional hazards regression analysis on the 7-lncRNA signature and clinical features in the training and independent cohorts, respectively. The results show that 7-lncRNA signature and TNM stage are independent prognostic factors for OS and DFS of patients in both the training (Table 3) and independent (Table 4) cohorts. The hazard ratio (HR) for tumor-related death in patients with high risk was about 2.5 times higher than those with low risk. Furthermore, we analyzed the prognostic role of the 7-lncRNA signature in all ESCC patients of the two cohorts. The result also exhibits that the 7-lncRNA signature is an independent prognostic factor for OS and DFS in all ESCC patients of the two cohorts (Additional file 1: Table S3). To further confirm that the 7-lncRNA signature is an prognostic predictor independent from TNM staging system, we analyzed the effect of 7-lncRNA signature on survival in different TNM stages in the training cohort. Since the number of patients with stage I is too small, we deleted these patients from the subsequent analysis. The results demonstrate that 7-lncRNA signature is a significant predictor for OS and DFS in patients with TNM stage II or III in the training cohort (Fig. 2), respectively, demonstrating that this signature is a prognostic factor independent of clinical stage and can offer additional information to evaluate survival of ESCC patients.

**Table 3:** Univariate and multivariable Cox analysis of the effects of 7-lncRNA signature and clinical characteristics on overall survival in the training cohort and independent cohort

Variables	Univariable analysis		Multivariable analysis	
	HR (95% CI)	p value	HR (95% CI)	p value
Training cohort				
Age (≤ 59 vs > 59)	1.48 (0.93–2.37)	0.101		
Gender (Male vs female)	1.04 (0.60–1.82)	0.878		
Tobacco use (Y vs N)	1.08 (0.66–1.78)	0.752		
Alcohol use (Y vs N)	1.01 (0.63–1.62)	0.981		
Tumor location (U vs M vs L)	1.41 (0.94–2.41)	0.095		
Differentiation (H vs Mt vs L)	1.34 (0.94–1.89)	0.104		
TNM stage (III vs I, II)	3.17 (1.94–5.20)	< 0.001	2.80 (1.80–4.61)	< 0.001
7-LncRNA signature (H-risk vs L- risk)	3.54 (2.11–5.92)	< 0.001	3.16 (1.90–5.31)	< 0.001
Independent cohort				
Age (≤ 59 vs > 59)	1.02 (0.62–1.70)	0.926		
Gender (Male vs female)	1.22 (0.70–2.12)	0.486		
Tobacco use (Y vs N)	1.12 (0.65–1.92)	0.682		
Alcohol use (Y vs N)	0.96 (0.57–1.61)	0.864		
Tumor location (U vs M vs L)	0.94 (0.60–1.47)	0.784		
Differentiation (H, Mt vs L)	1.40 (0.97–2.01)	0.069		
TNM stage (III vs I, II)	3.25 (1.94–5.46)	< 0.001	3.08 (1.83–5.18)	< 0.001
7-LncRNA signature (H-risk vs L-risk)	2.67 (1.55–4.58)	< 0.001	2.51 (1.46–4.33)	0.001

*Y* yes, *N* no, *U* upper, *M* middle, *L* low, *H* high, *Mt* moderate, *H-risk* high-risk, *L-risk* low-risk

**Table 4:** Univariate and multivariable Cox analysis of the effects of 7-lncRNA signature and clinical characteristics on disease-free survival in the independent cohort

Variables	Univariable analysis		Multivariable analysis	
	HR (95% CI)	p value	HR (95% CI)	p value
Training cohort				
Age (≤ 59 vs > 59)	0.96 (0.65–1.48)	0.906		
Gender (Male vs female)	0.78 (0.46–1.32)	0.357		
Tobacco use (Y vs N)	1.30 (0.82–2.05)	0.265		
Alcohol use (Y vs N)	1.10 (0.73–1.67)	0.657		
Tumor location (U vs M vs L)	1.26 (0.86–1.85)	0.238		
Differentiation (H vs Mt vs L)	2.49 (1.63–3.81)	< 0.001	2.23 (1.45–3.44)	< 0.001
TNM stage (III vs I, II)	2.41 (1.57–3.70)	< 0.001	1.93 (1.24–3.01)	0.003
LncRNA signature (H-risk vs L- risk)	2.36 (1.41–3.95)	< 0.001	2.28 (1.36–3.82)	0.002
Independent cohort				
Age (≤ 59 vs > 59)	1.04 (0.64–1.70)	0.868		
Gender (Male vs female)	1.14 (0.67–1.95)	0.632		
Tobacco use (Y vs N)	1.03 (0.62–1.73)	0.901		
Alcohol use (Y vs N)	1.05 (0.63–1.73)	0.858		
Tumor location (U vs M vs L)	0.98 (0.64–1.51)	0.941		
Differentiation (H vs Mt vs Low)	1.33 (0.94–1.89)	0.104		
TNM stage (III vs I, II)	2.66 (1.60–4.40)	< 0.001	2.58 (1.55–4.27)	< 0.001
LncRNA signature (H-risk vs L-risk)	2.36 (1.41–3.95)	0.001	2.28 (1.36–3.82)	0.002

*Y* yes, *N* no, *U* upper, *M* middle, *L* low, *H* high, *Mt* moderate, *H-risk* high-risk, *L-risk* low-risk

### The 7-lncRNA signature is able to improve the prognostic power of the TNM staging system in ESCC patients

**Fig. 3:**
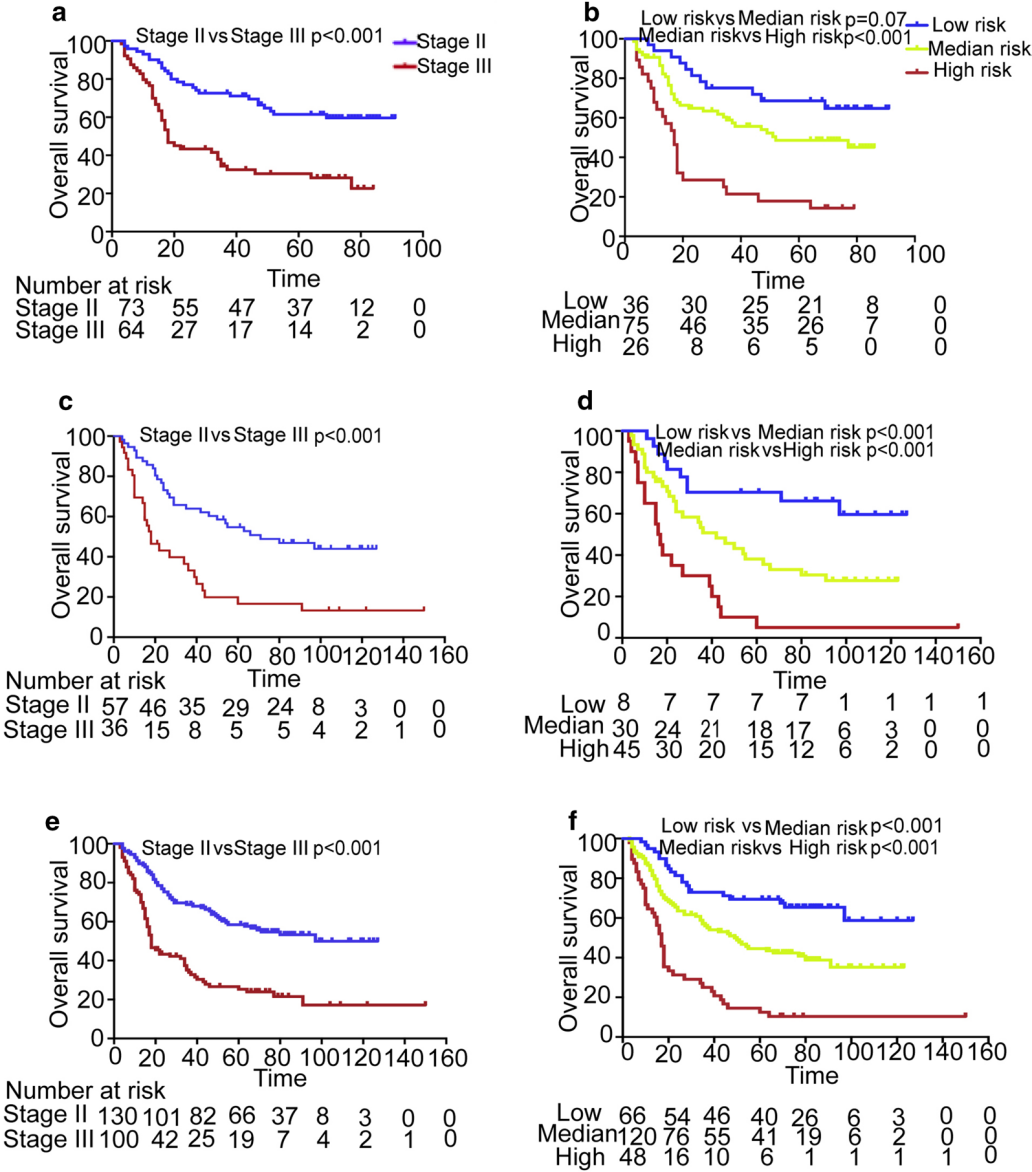
The 7-lncRNA signature improves survival prediction of TNM staging system in ESCC patients. The ESCC patients were defined as low-, moderate- and high-risk by the combination model of 7-lncRNA signature and TNM staging system, and then the survivals of these patients were analyzed with Kaplan–Meier curves and compared with the survivals predicted by TNM staging system. **a** Overall survival (OS) of patients with TNM stage II or III in the training cohort; **b** OS of patients with low-, moderate- or high-risk score in the training cohort. **c** OS of patients with TNM stage II or III in the independent cohort; **d** OS of patients with low-, moderate- or high-risk score in the independent cohort; **e** OS of patients with TNM stage II or III in the combination of two cohorts. **f** OS of patients with low-, moderate- or high-risk score in combination of two cohorts. In the Kaplan–Meier survival curves, the survival time unit is months

TNM staging system is a standard method for predicting survival in patients with malignant tumors and determining the treatment strategy. However, TNM staging system is not satisfactory enough to predict outcomes in ESCC patients. As mentioned above, ESCC patients with the same M0 stage may have significantly different prognoses [6]. Therefore, we tried to improve the prognostic power of TNM staging system in ESCC patients by incorporating the 7-lncRNA signature. For the same reason aforementioned, we removed the patients with stage I from this analysis. The combined risk score model was established with the following method: the signature score (low risk score = 0, and high risk score = 1) plus the TNM stage score (stage II = 1, stage III = 2). With this combined risk score model, patients were classified to a low- (1 score), moderate- (2 score) and high-risk (3 score) groups based on the combined risk score of every patients. The Kaplan–Meier survival analysis reveals that the patients with low-, medium- or high-risk have distinctly different OS (Fig. 3) and DFS (Additional file 2: Fig. S2) in the training cohort, independent cohort and combined cohort, indicating that this combined risk model can provide more accurate survival prediction with three different risk levels.

**Fig. 4:**
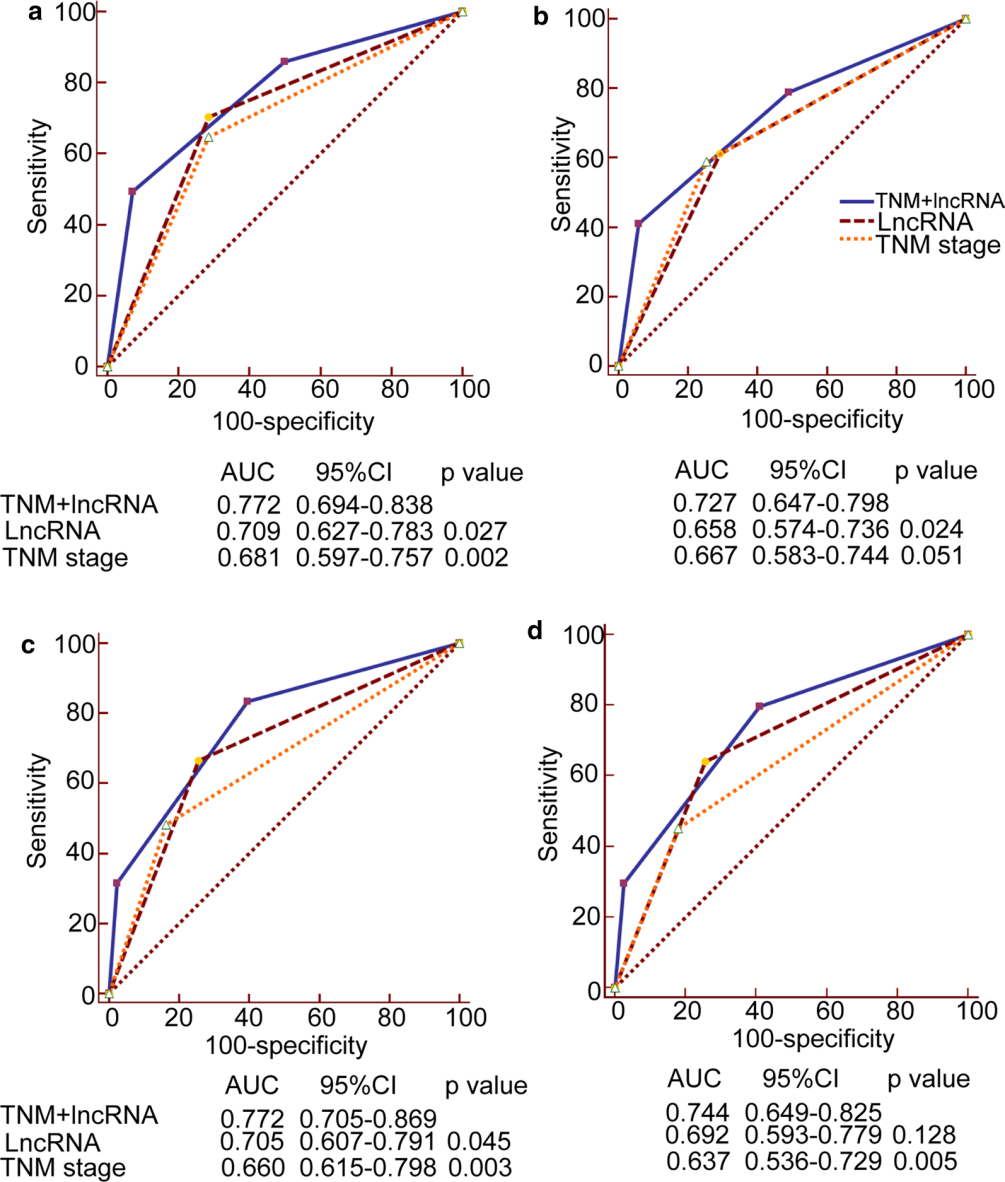
Comparisons of the performances of survival predictions made by the 7-lncRNA signature, TNM stage and combined model of the signature and TNM stage. The performances of survival predictions made by the three methods were compared using receiver operating characteristic (ROC) analysis. **a** ROC curves of the 7-lncRNA signature, TNM stage and combined model for overall survival (OS) prediction in the training cohort. **b** ROC curves of the three methods for disease-free survival (DFS) prediction in the training cohort. **c** ROC curves of the three methods for OS prediction in the independent cohort. **d** ROC curves of the three methods for DFS prediction in the independent cohort

To compare the performance of the combined risk score model with that of TNM staging system and 7-lncRNA signature, we performed receiver operating characteristic (ROC) analysis. The result reveals that the combined score model has better predictive performance in predicting OS (AUC: 0.772) and DFS (AUC: 0.727) than the 7-lncRNA signature (OS AUC: 0.709; DFS AUC: 0.658) and TNM stage alone (OS AUC: 0.681; DFS AUC: 0.667) in the training cohort (Fig. 4a, b). In the independent cohorts, we obtained similar results (Fig. 4c, d). We got much better predictive performance of the combined score model when the two cohorts were combined (Additional file 2: Fig. S3). These above results demonstrate that the 7-lncRNA signature can provide additional prognostic information to clinician and improve the predictive performance of TNM staging system in the evaluation of survival of ESCC patients.

### The 7 lncRNAs of the signature is likely to correlate with tumour-associated biological functions in ESCC

**Fig. 5:**
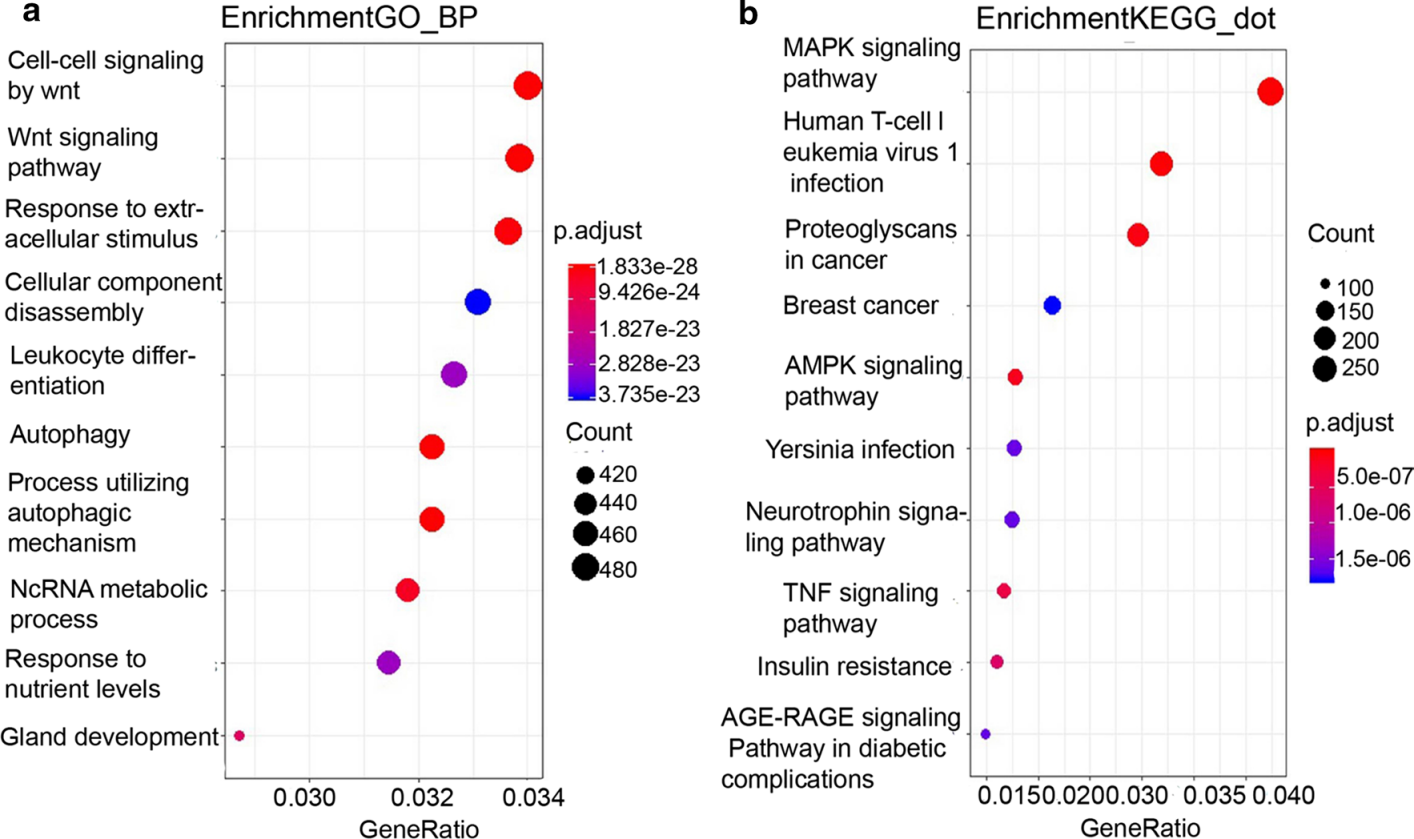
The 7-lncRNA signature is likely to correlate with tumour-associated biological processes. The target genes are regulated by transcription factors that could be bound by the 7 lncRNAs, and GO enrichment analysis and the KEGG pathway were performed on these genes. **a** The genes are enriched in the cell processes in GO enrichment analysis. **b** The genes are involved in the pathways in the KEGG pathway analysis

Finally, we hope to explore the potential role of the seven lncRNAs of this signature in ESCC development and progression. In our study, we could not conduct the lncRNA-gene interaction network because of lack of co-expressed lncRNA-mRNA data in ESCC. As we know, however, lncRNA can bind to transcription factors (TF) and regulate their transcriptional activity. To this end, we identified that 44 TFs could be bound by the 7 lncRNAs in the public lncRNA-TF interactome database RegRNA2.0, and 17,362 target genes can be regulated by the 44 TFs in Harmonizome database. Then, we found that 555 of 17,362 genes were overlapped with 575 enriched genes in esophageal cancer obtained from TCGA database as shown in Venn diagram (Additional file 2: Fig S4). Next, we conducted GO enrichment analysis and the KEGG pathway. In the GO enrichment analysis, these genes are mainly enriched in the cell–cell signaling by Wnt, Wnt signaling pathway and autophagy (Fig. 5a). In the KEGG pathway analysis, the 7 lncRNAs are primarily involved in MAPK signaling pathway, proteoglycans in cancer, and AMPK signaling pathway (Fig. 5b). These results suggest that the lncRNAs signature is not only a prognostic factor but also likely regulates the cancer-related pathways in the progression of esophageal cancer.

## Discussion

As the standard method for predicting survival in human cancer, TNM staging system plays a critical role in treatment choice for ESCC patients. TNM staging system is based on the size of primary cancer and anatomical site of the primary and metastatic cancers. However, malignant behavior is mainly determined by the molecular and genetic characteristics of the cancer. Consequently, survival prediction solely based on the tumor size and anatomical site is not fully satisfactory yet to physicians in clinical practice [30, 31], suggesting that evaluation of prognosis should be based on both the TNM stage system and molecular/genetic characteristics of the cancer. Gene expression profile is one of the most comprehensive ways in understanding the molecular changes in cancer. In recent decades, gene expression profiles have been explored in nearly all types of cancer due to advancements in high throughput technologies (microarray, deep-sequencing, PCR and others), and many gene signatures have been identified for predicting survival of patients with cancer [32–34]. For example, a 70-gene signature has been used for predicting patients’ survival of in breast cancer [35] and a 42-gene signature has been used for prediction of disease relapse in early stage colon cancer [36]. In esophageal cancer, many studies on gene expression profile have been reported, but the case number in these studies is small (the biggest being 89 cases) [37] and no gene signature has been applied in this disease at present. Recently, non-coding RNA (including microRNA [miRNA], long non-coding RNA and others) has been found to play critical roles in physiological and pathological processes of organisms [38–40], and involved in disease processes including that of cancer. In previous study, we profiled the microRNA expression of nasopharyngeal carcinoma (NPC) and hepatocellular carcinoma (HCC) using a custom microarray and identified a 5-miRNA signature in NPC [41] and 20-miRNA signature in HCC [26], which were independent prognostic factors and could predict survival and metastasis of patients with NPC and HCC, demonstrating that the two signatures can add more prognostic value to the TNM staging system. In literature reports, lncRNA expression signatures can predict survival in patients with lung adenocarcinoma, colorectal cancer and hepatocellular carcinoma [42–44]. In ESCC patients, although many studies on lncRNA expression profiles have been reported, most studies only employed a few paired cancer and non-cancer samples [45, 46], and only one 3-lncRNA prognostic signature has been identified in 119 esophageal cancers and paired non-cancerous tissues with microarray method [24]. In the present study, we detected 141 ESCC samples with the custom lncRNA microarray and identified a 7-lncRNA signature for predicting survival of patients with ESCC in the training cohort, and validated it with RT-PCR in the independent cohort, suggesting that this 7-lncRNA signature is reliable. We then demonstrated that this signature is an independent prognostic factor with Cox regression analysis and can predict distinct survivals in the patients with the same clinical stage, implying that this signature can provide additional information in predicting survival of ESCC patients. As we expected, our result showed that this 7-lncRNA signature significantly improved the prognostic power of TNM staging system when it combined with TNM stage (Fig. 4), indicating that this signature will be a useful biomarker for predicting survival in ESCC patients. However, this study has limitations: firstly, the custom microarray only includes 2412 lncRNAs, and as a consequence, this signature may not be the best predictor for survival in ESCC; secondly, this study was conducted at a single cancer center and the signature should be further validated in patients from different geographical areas. Nevertheless, our study warrants a multi-center study to further validate the signature’s utility and the study of functions and mechanisms of these 7 lncRNAs.

## Conclusions

In this study, we identified a 7-lncRNA signature that can predict the prognosis in patients with ESCC and may help in determining treatment when combined with the TNM stage system.

## Supplementary information

**Additional file 1.** Additional tables.

**Additional file 2:** Additional figures.

## Data Availability

All data in our study are available upon request.

## Abbreviations

ESCC: esophageal squamous cell carcinoma
lncRNAs: long non-coding RNAs
TNM: tumor-node-metastasis
OS: overall survival
DFS: disease-free survival
ROC: receiver operating characteristic
AUC: false discovery rate
qRT-PCR: quantitative real-time reverse transcription polymerase chain reaction
HR: hazard ratio
95% CI: 95% confidence interval
SAM: significance analysis of microarray
TF: transcription factor
GO: Gene Ontology
KEGG: Kyoto Encyclopedia of Genes and Genomes
