# Supplementary material for: The prognostic value of a seven-lncRNA signature in patients with esophageal squamous cell carcinoma: a lncRNA expression analysis
Source: J Transl Med. 2020 Jan 31;18:47. doi: 10.1186/s12967-020-02224-z (PMC6995134; doi:10.1186/s12967-020-02224-z)
Supplement: Supplementary file 1 — Additional file 1. Additional tables. [file 12967_2020_2224_MOESM1_ESM.docx]

**Additional File 1: Tables**

**Table S1.The primer information of lncRNAs**.

|  |  | **Forward primer** |  | **Reverse primer** |
| --- | --- | --- | --- | --- |
| XLOC_001061 |  | ACCACCTTCTTGTCTGCCCA |  | GAACAACGCATTCTTGCCTCC |
| RP11-473M20.9 |  | CCCAACTGTCACGAGCAACAC |  | CTTTCGCCCTGTTGGTCAGTG |
| BQ376030 |  | AGAAGATGCACGCCACCCTAG |  | ATGGAGTGCCCGTGGACTTC |
| ASLNC11164 |  | ATCTCCGAATGGACCTCAGC |  | CTCCAATGCCCCTTTTCAGG |
| BF894811 |  | GGAAACAGCCATCAGGAGCC |  | TCTGGGATCGTTGTCCTTTGC |
| XLOC_007869 |  | ATCTTTGTGGCTGCGGGAGT |  | GCTGAGGGCCAGAACATACC |
| CK327190 |  | GGAAACAGCCATCAGGAGCC |  | TCTGGGATCGTTGTCCTTTGC |
| XLOC_006476 |  | TACAGGCATGAGCCACTGCG |  | TGAGGAGGAGGAGGAGAAGG |
| AC007131 |  | GAAGCATGTTTGGGCTGAGAGG |  | GTCTGCACTCCTACTGGGCATT |

**Table S2. Summary of 16 lncRNAs associated with overall survival of ESCC patients in the training cohort**

| **NO** | **lncRNA** | **Weight** | **P value** | **HR(95%CI)** | **Putative function** |
| --- | --- | --- | --- | --- | --- |
| **1** | **XLOC_007869** | **-0.182** | **0.051** | **0.694-1.001** | **Protective** |
| 2 | XLOC_003292 | -0.178 | 0.025 | 0.716-0.978 | Protective |
| **3** | **CK327190** | **-0.172** | **0.035** | **0.718-0.988** | **Protective** |
| **4** | **XLOC_006476** | **-0.150** | **0.081** | **0.727-1.019** | **Protective** |
| 5 | XLOC_012786 | -0.145 | 0.034 | 0.756-0.989 | Protective |
| **6** | **ASLNC11164** | **0.112** | **0.088** | **0.983-1.273** | **High-risk** |
| 7 | BF926453 | 0.118 | 0.042 | 1.004-1.26 | High-risk |
| **8** | **BF894811** | **0.125** | **0.027** | **1.014-1.265** | **High-risk** |
| 9 | ASLNC12986 | 0.148 | 0.085 | 0.980-1.372 | High-risk |
| **10** | **BQ376030** | **0.155** | **0.037** | **1.009-1.35** | **High-risk** |
| 11 | ASLNC09671 | 0.168 | 0.020 | 1.027-1.363 | High-risk |
| **12** | **RP11-473M20.9** | **0.191** | **0.038** | **1.011-1.45** | **High-risk** |
| 13 | ASLNC17843 | 0.192 | 0.071 | 0.983-1.492 | High-risk |
| 14 | ASLNC21872 | 0.195 | 0.089 | 0.971-1.521 | High-risk |
| 15 | ASLNC03344 | 0.307 | 0.076 | 0.969-1.909 | High-risk |
| 16 | ASLNC07601 | 0.386 | 0.025 | 1.049-2.063 | High-risk |

The lncRNAs with bold font are the ones of 7-lncRNA signature.

**Table S3. Univariate and multivariable Cox regression analysis of the effects of 7-lncRNA signature and TNM stage on overall survival and disease-free survival in all ESCC patients.**

| **Variables** | **Univariable analysis** | | **Multivariable analysis** | |
| --- | --- | --- | --- | --- |
|  | **HR(95%CI)** | **P value** | **HR(95%CI)** | **P value** |
| **Overall survival** |  |  |  |  |
| TNM stage (IV,III vs I,II) | 3.03 (2.13-4.31) | <0.001 | 2.86 (2.00-4.07) | <0.001 |
| LncRNA signature (H-risk vs L-risk) | 3.12 (2.15-4.53) | <0.001 | 2.95 (2.03-4.29) | <0.001 |
| **Disease-free survival** |  |  |  |  |
| TNM stage (IV,III vs I,II) | 2.53 (1.84-3.50) | <0.001 | 2.430 (1.76-3.36) | <0.001 |
| LncRNA signature ( H-risk vs L-risk) | 2.41 (1.74-3.35) | <0.001 | 2.309 (1.66-3.21) | <0.001 |
