# Supplementary material for: The prognostic value of a seven-lncRNA signature in patients with esophageal squamous cell carcinoma: a lncRNA expression analysis
Source: J Transl Med. 2020 Jan 31;18:47. doi: 10.1186/s12967-020-02224-z (PMC6995134; doi:10.1186/s12967-020-02224-z)
Supplement: Supplementary file 2 — Additional file 2: Additional figures. [file 12967_2020_2224_MOESM2_ESM.docx]

**Additional File 2: Figures**


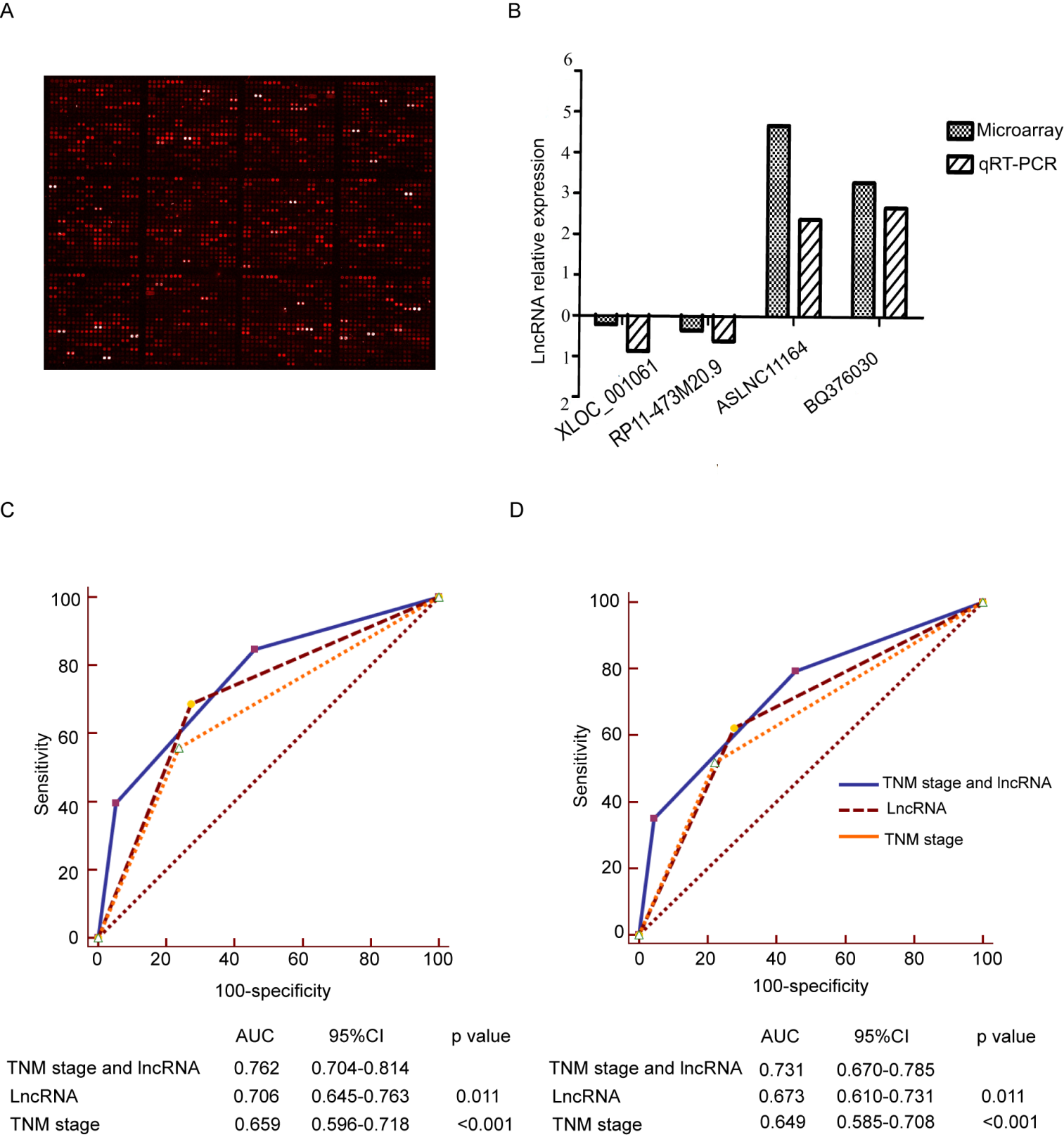


**Figure S1. Microarray and qRT-PCR detection of lncRNA expression.** The custom microarray was used to detect lncRNA expression profiles of ESCC samples in the training cohort, and qRT-PCR was employed to measure the expression of the selected four lncRNAs to confirm the expression levels of lncRNAs detected by microarray. **(A)** The scanning images of lncRNA microarray hybridized by total RNA. (B) The comparison of microarray data and qRT-PCR data in the training cohort.

**
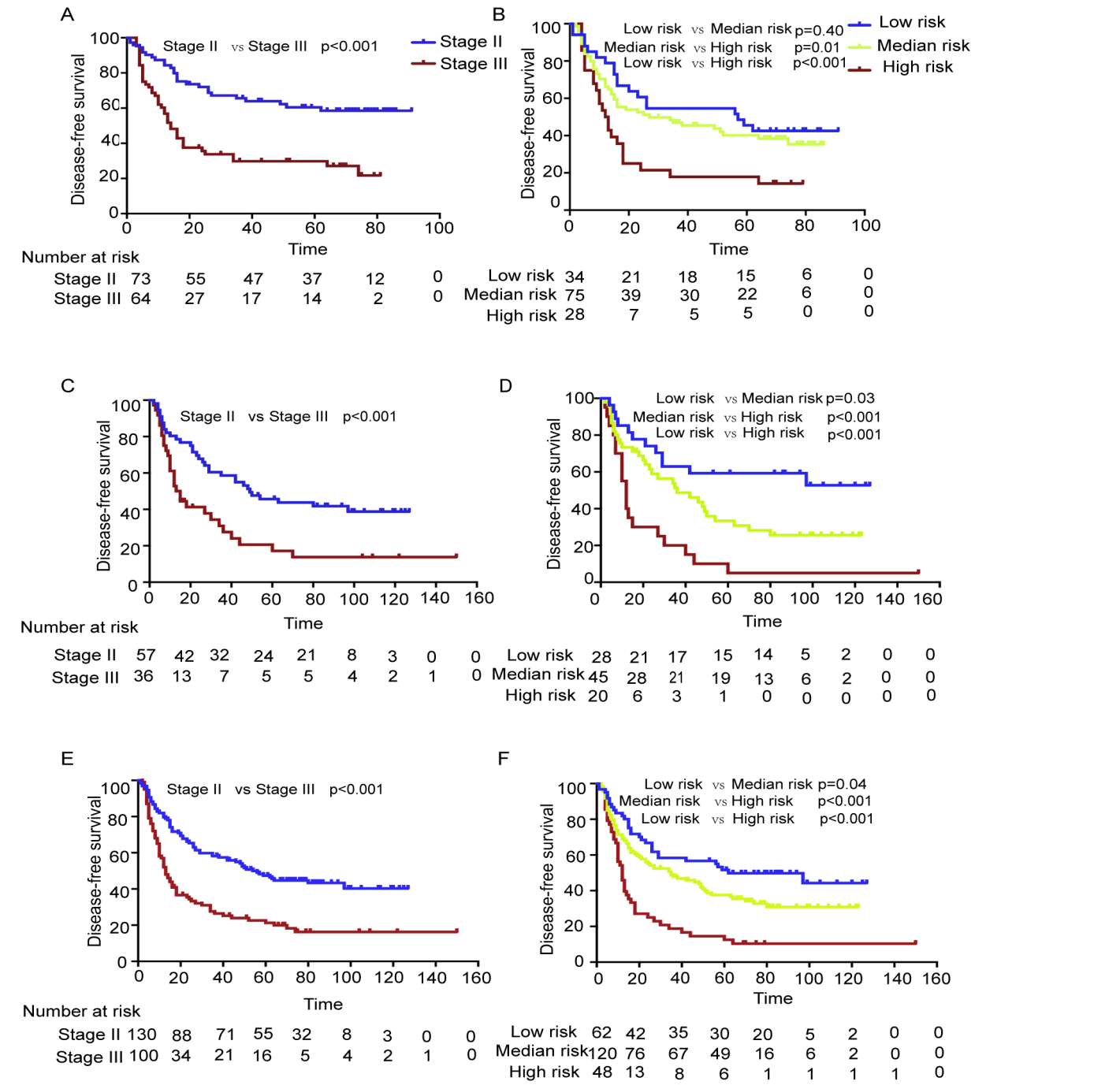
 Figure S2. Kaplan-Meier survival analysis of Disease-free survival (DFS) of ESCC patients predicted by TNM staging system or combined risk model of the 7-lncRNA signature and TNM staging system.** **(A)** DFS of patients with TNM stage II or III in the training cohort. **(B)** DFS of patients with low-, medium- or high-risk defined by the combined risk model in the training cohort. **(C)** DFS of patients with TNM stage II or III in the independent cohort. **(D)** DFS of patients with low-, medium- or high-risk in the independent cohort. **(E)** DFS of patients with TNM stage II or III in the combination of the two cohorts. **(F)** DFS of patients with low-, medium- or high-risk in the combination of the two cohorts.

A B


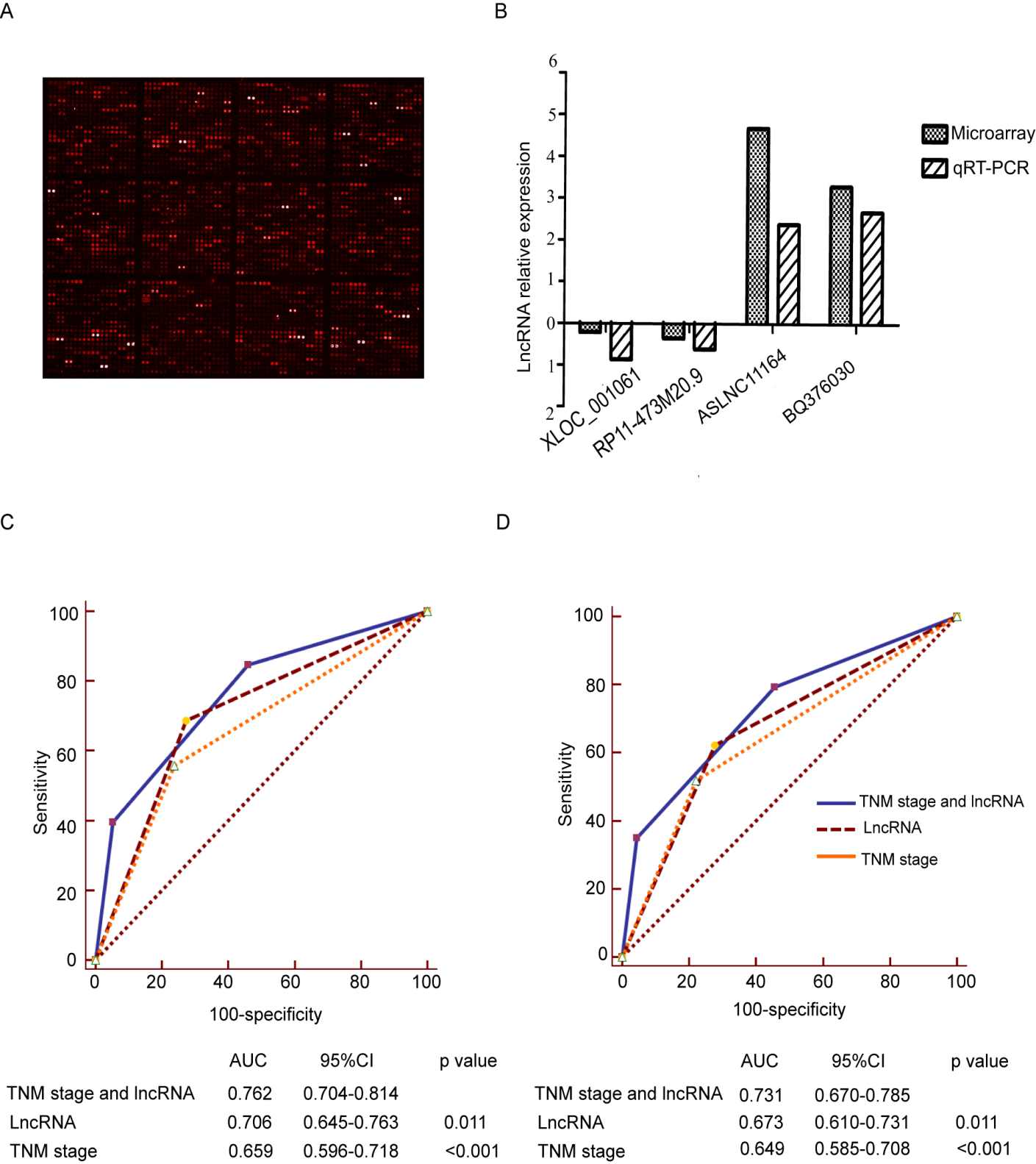


**Figure S3.** **Comparisons of the** **performances of** **survival predictions made by** **the 7-lncRNA signature, TNM stage and combined model of the signature and TNM stage in all ESCC patients.** The performances of survival predictions made by the three methods were compared using receiver operating characteristic (ROC) analysis. **(A)** ROC curves of the 7-lncRNA signature, TNM stage and combined model for overall survival (OS) prediction in all ESCC patients of the two cohort. **(B)** ROC curves of the three methods for disease-free survival (DFS) prediction in all ESCC patients of the two cohorts.

**
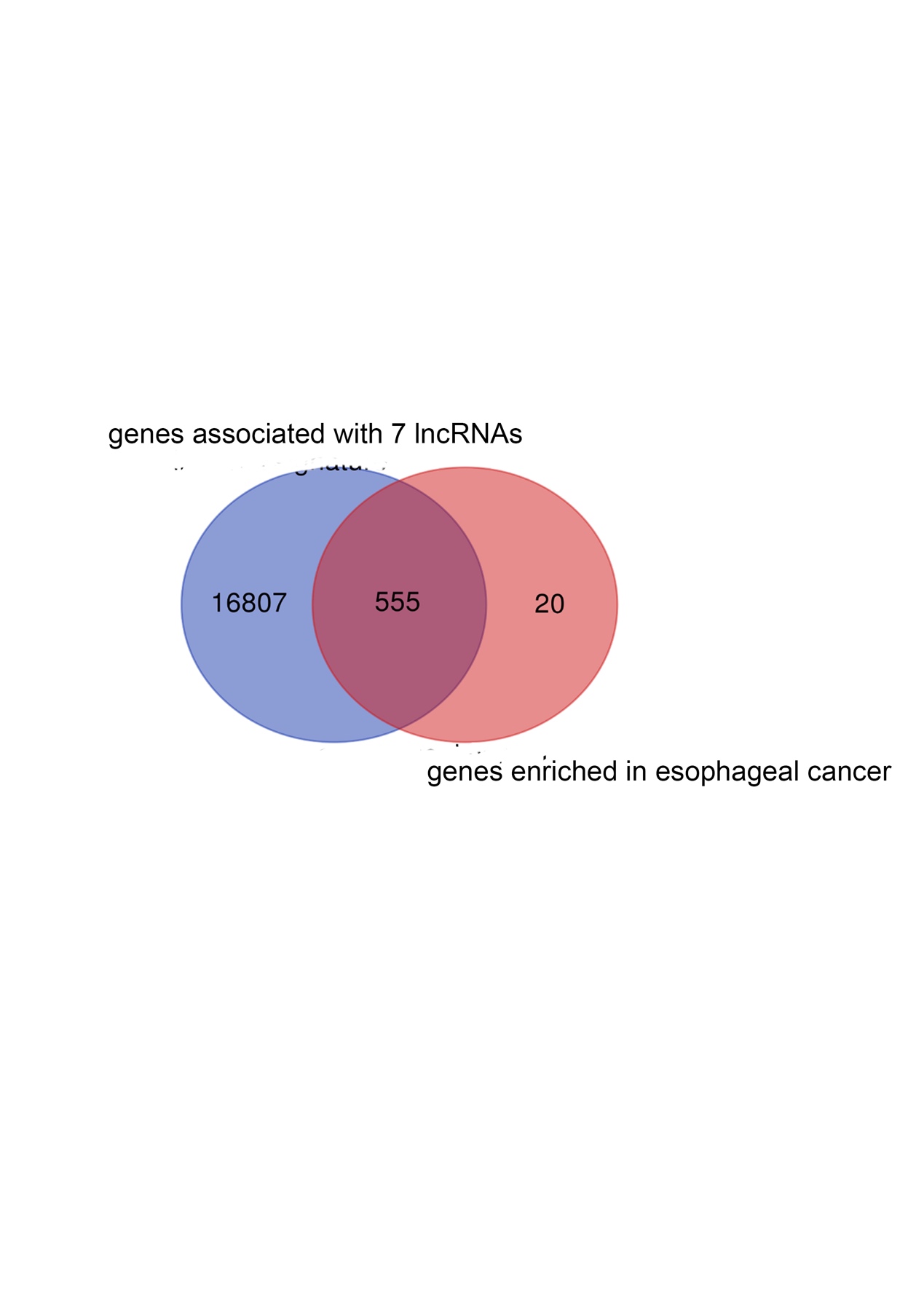
**

**Figure S4.** **The genes associated with 7 lncRNAs in esophageal cancer.** Venn diagram is used to exhibited that the target genes regulated by 44 transcription factors associated with 7 lncRNAs are overlapped with the genes enriched in esophageal carcinoma obtained from TCGA database.
